# Road Killed Carnivores Illustrate the Status of Zoonotic Helminthes in Caspian Sea Littoral of Iran

**Published:** 2017

**Authors:** Aida VAFAE ESLAHI, Eshrat Beigom KIA, Iraj MOBEDI, Meysam SHARIFDINI, Milad BADRI, Gholamreza MOWLAVI

**Affiliations:** 1. Dept. of Medical Parasitology and Mycology, School of Public Health, Tehran University of Medical Sciences, Tehran, Iran; 2. Center for Research of Endemic Parasites of Iran (CREPI), Tehran University of Medical Sciences, Tehran, Iran; 3. Dept. of Medical Microbiology, School of Medicine, Guilan University of Medical Sciences, Rasht, Iran; 4. Dept. of Medical Parasitology, School of Medical Sciences, Tarbiat Modares University, Tehran, Iran

**Keywords:** Carnivore carcasses, Zoonotic helminthes, Iran

## Abstract

**Background:**

Carnivore carcasses on the roads can be regarded as study materials in parasitology and eco-epidemiology. Stray carnivores such as dogs and cats are known to harbor so many different pathogens like zoonotic helminthes. The current investigation, apparent the status of the helminthic parasites found in road killed carnivores from different parts of Guilan Province north of Iran.

**Methods:**

Fifty road killed carnivores including 27 stray dogs (*Canis familiaris*), 11 golden jackals (*Canis aureus*) and 12 stray cats (*Felis catus*) were collected from 21 locations of Guilan Province, during Apr to Nov 2015. Internal organs of the carcasses, including digestive tract, heart, kidneys, lungs, liver, skin, eyes as well as muscles were carefully inspected and sampled for helminthological investigation.

**Results:**

About 80% of the 50 carnivores, (stray dogs 77.77%, golden jackals 81.81%, and stray cats 91.66%) were found naturally infected with helminthic parasites. *Dipylidum caninum*, *Toxocara cati*, *Toxocara canis*, *Toxascaris leonine*, *Ancylostoma caninum*, *Ancylostoma tubaeforme*, *Dirofilaria immitis*, *Dioctophyma renale*, *Dipylidum caninum*, *Echinococcus granulosus*, Mesocestoides spp*., Taenia* hydatigena, *Taenia hydatigera*, *Joyuxiella* spp.*, Spirometra* spp. are reported herein.

**Conclusion:**

The prevalent occurrence of zoonotic helminthes such as *T. canis*, *T. cati*, *T. leonina*, *E. granulosus*, *D. immitis* and *D. renale* in stray carnivores should be considered as a public health hazard, specifically within a vast tourism area like Guilan Province.

## Introduction

Transmission of the zoonotic helminthes of carnivores to human populations may frequently occur worldwide (1). The importance of parasites anchoring in diverse species of stray canids and felids has been well emphasized and recorded throughout the literature so far. Dogs and cats as two common stray carnivores are known to carry different pathogens like zoonotic helminthes in the human residing environments (2). Carnivore carcasses on the roads can not only provide study materials for parasitological investigations, with no aggressive action to the wildlife but also are regarded as advantageously available samples in eco-epidemiological studies (3). Meanwhile, exploring some prevalent parasitic agents in the world like *Toxocara* spp., *Echinococcus granulosus*, *Trichinella* spp., *Ancylostoma* spp., and *Dirofilaria* spp. can be performed in this manner. The world public health challenging issues, visceral larva migrans (VLM) due to ascarids of dogs and cats as well as the accidental occurrence of human dirofilariasis, are also exemplary herein (4–7). Concerning dramatical reduction of cystic echinococcosis in previous decades, the prevalence of hydatidosis due to *E. granulosus*, harboring by canids like stray dogs is still regarded as a global health concern (8). In Iran however, echinococcosis known as a prevalent animal zoonotic infection and to some extent in humans, can be surveyed traced in road killed carcasses (9). Guilan Province, in north Iran, due to having temperate climate and broadleaf forests has provided a suitable shelter place for different kinds of mammals. Road accidents killing carnivores within the region is also frequent mostly at night due to the heavy traffic and fast driving cars in connecting touristic roads. In current study, we took advantage of available carnivore carcasses to estimate the status of the helminthic parasites, especially zoonotic species in Guilan Province.

## Materials and Methods

### Study area

Guilan Province is located on the southern shores of the Caspian Sea about 14042 km^2^, north of Iran. Alborz mountains have embraced southern parts of the province provided further diversity to the area added to the Caspian climatological situation closing the condition to Mediterranean-like climate (10). The area “37. 2774 N 49. 5890 E” has a temperate climate and abundant annual rainfall with 1359 mm in volume. Regarding the current research objectives, the frequent movement of carnivores likely canids as well as stray dogs and cats on connecting roads in the region that may cause the loss of some these animals continually is considerable.

### Sample Collection

**Fig. 1:**
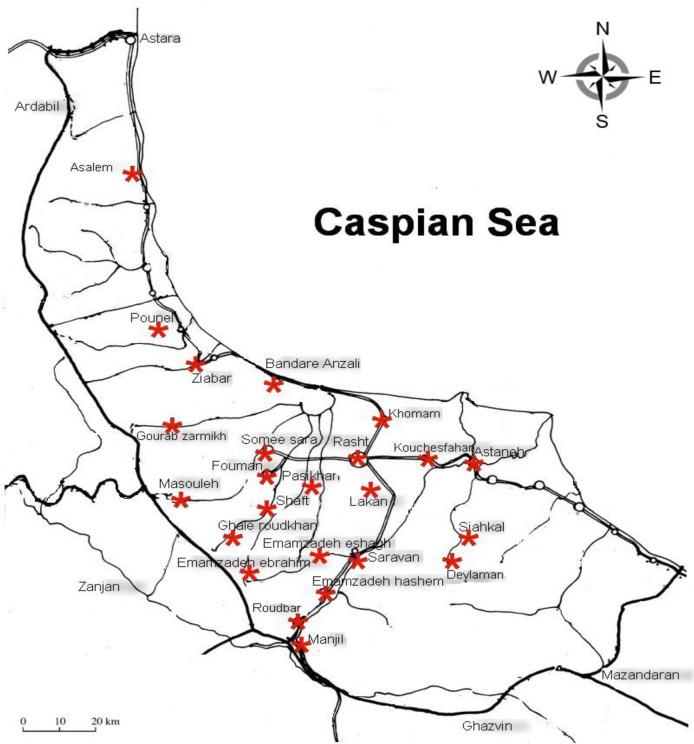
The collecting locations of road killed carcasses in Guilan Province

During Apr to Nov 2015, 50 road killed carnivores including 27 stray dogs (*Canis familiaris*), 11 golden jackals (*Canis aureus*) and 12 stray cats (*Felis catus*) were collected from 21 locations in Rasht, Anzali Port, Roudbar and Deylaman (Fig. 1).

Internal organs including digestive tract, heart, kidneys, lungs, liver, skin, eye, and muscles of the carcasses were initially, removed, isolated, labeled and transferred to the laboratory for complete dissection process. In the laboratory, large and small intestines were slit-ten open and the lumens were crashed well, up to the muscular layer and were thoroughly detected for parasite by different magnifications using binocular and microscope. Meanwhile, examination for detecting *Trichinella* larvae was also performed on mussel necropsy specimen, for each carcass.

### Helminth collection

The contents of scraped epithelium of small intestine were studied cautiously in 10 cm plates, entirely to the latest drop.

Recovered helminthes were stored in 70-degree ethanol while the specimen condition aiming to morphological identification such as body relaxation and flattening were done for cestodes, specifically. For tapeworms, carmine acid staining, and for transparency of nematodes lacthophenol solution were used. Classical identification for the helminthes has been finalized by the use of reliable key references (11–13).

In this study, ethical issues were not neglected due to avoiding of killing animals as research material. Animal carcasess found on the roads caused by traffic have been the source of sampling in this survey.

## Results

According to Table 1, 80% of the 50 carnivores, (Stray dogs 77.7%, golden jackals 81.8% and stray cats 91.6%) were found naturally infected with helminthic parasites.

**Table 1:** Prevalence of helminthic parasites recovered from the carnivores carcasses present study area

**Parasites**	**Dogs (27) (*Canis familiaris*)**	**Golden Jackals (11) (*Canis aureus*)**	**Cats (12) (*Felis catus*)**	**Total (85)**
**Nematodes**	**No (%)**	**No (%)**	**No (%)**	**46 (54.11%)**
*Toxocara canis*	5 (18.5)	3 (27.2)		8
*Toxocara cati*			9 (75)	9
*Toxascaris leonina*	4 (14.8)	2 (18.1)		6
*Ancylostoma caninum*	6 (22.2)	1 (9)		7
*Ancylostoma tubaeforme*			2 (16.6)	2
*Dirofilaria immitis*	7 (25.9)	1 (9)		8
*Dioctophyma renale*	5 (18.5)	1 (9)		6
	Cestodes			39 (45.88%)
*Dipylidium caninum*	9 (33.3)	3 (27.2)		12
*Echinococcus granulosus*	7 (25.9)	3 (27.2)		10
*Mesocestoides* spp.	2 (7.4)			2
*Taenia hydatigena*	5 (18.5)	2 (18.1)		7
*Taenia hydatigera*			1 (8.3)	1
*Joyuxiella* spp.	2 (7.4)			2
*Spirometra* spp.	2 (7.4)	3 (25)		5

**Fig. 2:**
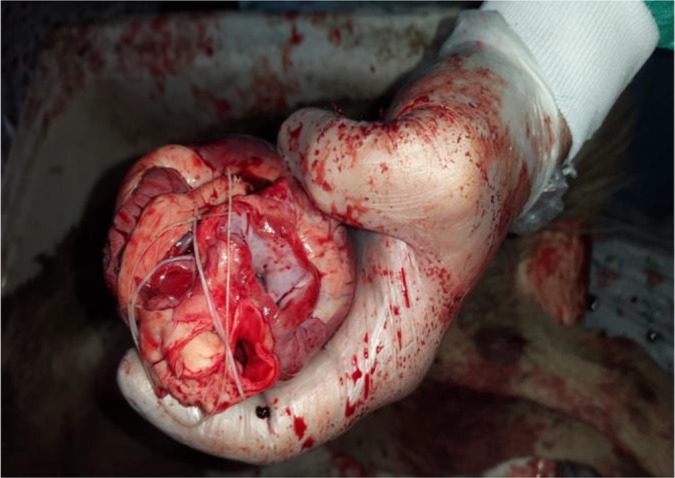
*Dirofilaria immitis* in heart of stray dog

The most prevalent helminthic parasite in stray dogs and stray cats was *Dipylidum caninum* (33.3%) and *Toxocara cati* (75%) respectively. In golden jackals, the prevalence of *Toxocara canis*, *D. caninum,* and *E. granulosus* were seen equal, 27.2%. *Toxascaris leonina* was found in 18.5% of stray dogs and 9.1% of golden jackals. In addition*,* 29.6% of stray dogs, and 9% of the golden jackals have been parasitized by *Dirofilaria immitis* (Fig. 2). The giant kidney worm*, Dioctophyma renale* was also detected in 14.8% of the stray dogs and 9% of the golden jackals. In this survey, mix infection was seen in 70.3% of stray dogs, 36.3% of golden jackals and 41. 6% of stray cats with the most co-infection of *D. renale* with *D. immitis* (7.4%) and *T. canis* with *Ancylostoma caninum* (11.1%) in dogs, *E. granulosus* with *Taenia hydatigena* (18.1%) in jackals and *Spirometra* spp. with *T. cati* (16.6%) in cats. Among the carcasses studied, *A. caninum* were seen in 22 cases.

Two percent of stray dogs and 9% of the golden jackals and *Ancylostoma tubaeforme* was detected in 16. 6% of the stray cats*.* The same occurrence of *Mesocestoides* spp. and *Joyuxiella* spp. 7.4%, were seen in stray dogs. In 29.6% of stray dogs, also *E. granulosus* was detected. Meanwhile, *Spirometra* spp. was found in 7.4% of the stray dogs and 33.3% of the stray cats. *Taenia hydatigera* was merely found in 8.3% of stray cats while *T. hydatigena* was removed from 14.8% of stray dogs and 18.1% of golden jackals. Moreover, co-infection between helminthic parasites calculated for present study is shown in Table 2. According to recorded data, the overall situation of mix infections are as, 70.3%, 36.3%, and 41.6% in stray dogs, golden jackals and stray cats, respectively.

**Table 2:** The co-infections of parasitic helminthes in stray dogs, golden jackals and stray cats

**Carnivores (n=50)**	**Co-infection of Helminthic parasites (%)**
Dog (n=27)	*D. immitis* with *D. renale* (7.4%) – *T. canis* with *A. caninum* (11.11%)
Golden jackal (n=11)	*E. granulosus* with *T. hydatigena* (18. 18%)
Cat (n=12)	*Spirometra* sp. with *T. cati* (16.66%)

The most co-infection occurrences were seen for *D. renale* & *D. immitis* (7.4%), *T. canis* & *A. caninum* (11.1%) in stray dogs, and that for *E. granulosus* & *T. hydatigena* in 18.1% of golden jakals*.* In 16.66% of stray cats also, co-infection were seen between *Spirometra* spp. & *T. canis*.

## Discussion

Road killed animals are found abundant on fast driving roads everywhere in the world. This source of samples investigated for zoonotic pathogens are considered valuable. From the medical and veterinary points of views, findings collected from road killed animal can be statistically attributed to other animal species in the region and circulate pathogens amongst predator-pray cycles (6, 14, 15). Meanwhile, the importance of carnivores as a source of 374 pathogenic agents attracts the minds of health authorities to estimate the situation of zoonoses and emerging infections in their surrounding environment (16). The share of wild carnivores in this interaction has however estimated noticeable in the literature (2). We took advantage of present opportunity to understand the status of transmittable zoonotic helminthes in Guilan Province, by means of road animal mortalities including stray dogs, jackals, and cats. In some previous works carried out in Iran (17–21) and in Caspian Sea littoral so far (22–24), similar findings as what is presented herein, have been reported. From the standpoint of zoonotic parasites, majority of the helminthes recovered in this work, are of great importance, with special regards on *D. immitis* and *D. renale*, with a prevalence of 25. 9% and 18. 5% in stray dog respectively and the rate of both parasites was 9% in golden jackals. Although a series of common zoonotic helminthes including *T. canis, T. cati, T. leonina* and *E. garanulosus* were identified in this survey, nevertheless, the finding of the rarer and light ones such as *D. caninum*, *Spirometra* spp. and *Mesocestoides* spp. In the region should be also taken into account. Meanwhile, reporting of *Mesocestoides* spp. in the current paper seems worth mentioning as the occurrence of 27 human cases, merely in the United States being regarded (25). The existence of *D. immitis* and *D. renale* in stray dogs and jackals in Guilan which our present results have lightened the issue should be seriously regarded by health and tourism sectors than the past. Concerning the background of trichinellosis in the wildlife in Iran already documented by others (26–28), the negative result of studied carcasses in present occasion is debating. This controversial conclusion regarding the existence of Trichinellosis in the wildlife of Guilan Province can be attributed the low larval burden in carcasses and /or inaccurate laboratory method used herein. Study of parasites fauna in road-killed carnivore in different parts of the world, would be a valuable source of information assisting the health programmers in prevention of some zoonotic infection transmission as well as the rare but harmful helminthiasis to humans in the given region.

## Conclusion

As Guilan Province is a tourism hub and considerable numbers of people from different parts of Iran, visiting this area, the transmission of zoonotic infections, especially helminthic parasites that stray carnivores such as dogs, cats and jackals are harboring them, is a public health danger. As regards to the observations of the current study, *D. immitis*, *D. renale*, *E. granulosus*, *Toxocara* spp. and *T. leonina* are the most important helminthic parasites transmitted to humans from the stray carnivores above. More parasitological and epidemiological studies should be performed to illustrate the situation of dissemination and transmission of this infectious to both animals and humans and the control and management of the program of the mentioned zoonotic parasites in this area with emphasis on public health is required.
